# β-Galactosidase: From lactose hydrolysis to glycosylation engineering

**DOI:** 10.1016/j.engmic.2026.100306

**Published:** 2026-09-21

**Authors:** Wei Wei, Jinshan Guo

**Affiliations:** Department of Biomaterials and Stem Cells, Suzhou Institute of Biomedical Engineering and Technology, Chinese Academy of Science, Suzhou, 215011, China

**Keywords:** β-galactosidase, Transglycosylation, Galactosylation, Lactose-derived oligosaccharides, Galactoside compounds

## Abstract

β-galactosidase is a naturally occurring and widely distributed glycoside hydrolase known for its ability to specifically hydrolyze β-glycosidic bonds in lactose and other oligosaccharides. Beyond its robust hydrolytic activity, the enzyme also exhibits superior transglycosylation activity, making it indispensable in the industrial production of galacto-oligosaccharides. Its transglycosylation capacity is further characterized by broad substrate specificity. This enzyme can also catalyze glycosylation of various carbohydrate and non‑carbohydrate acceptors, yielding several novel galactosylated products. These products often exhibit enhanced chemical/physical properties, such as improved solubility, stability, and bioactivity, compared to those of their non‑glycosylated precursors. This review summarizes the synthesis of lactose‑derived oligosaccharides by β-galactosidase, as well as the modification of diverse carbohydrate acceptors and non‑carbohydrate acceptors using β-galactosidases from different sources, highlighting their excellent transglycosylation performance. We also discuss the improvements in characteristics and the acquisition of new biological activities following glycosylation. Therefore, this review provides a useful reference for developing novel high‑performance glycosylated products and for promoting broader application of β-galactosidase in the food and pharmaceutical industries.

## Graphical abstract

**Figure:**
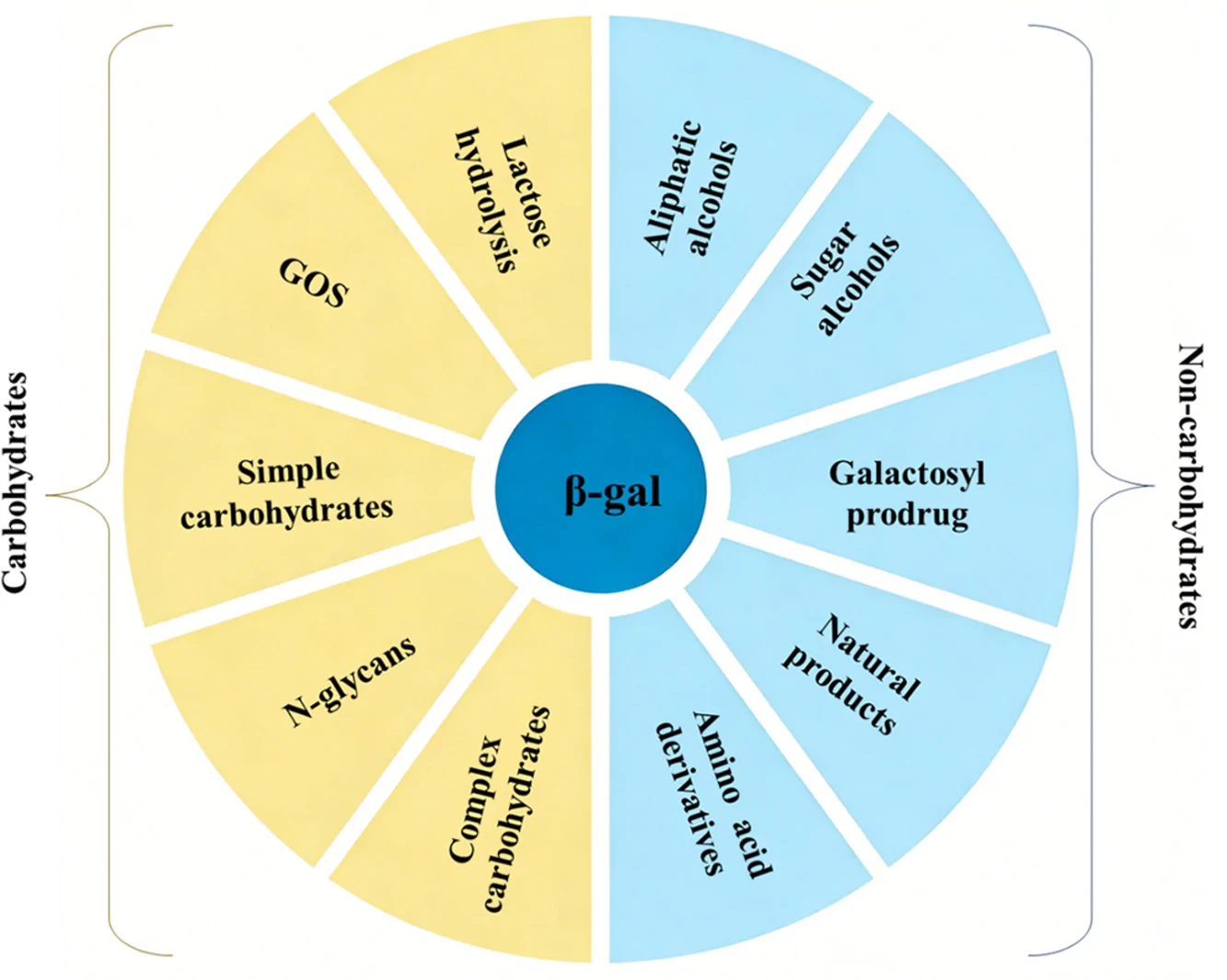

## 1 Introduction

β-Galactosidase (EC 3.2.1.23, β-gal), commonly known as lactase, primarily catalyzes the hydrolysis of lactose into galactose and glucose. This biochemical process has been widely harnessed in the food industry to produce low-lactose dairy products, thereby improving their digestibility, sweetness, solubility, and flavor. The widespread application of this enzymatic process has significantly alleviated the global health issue of lactose intolerance [1,2]. In the dairy industry, β-gal is routinely used in milk processing to produce low-lactose or lactose-free milk products. By hydrolyzing lactose prior to consumption, the enzyme effectively reduces the lactose content of the milk to <0.1% (w/v) to yield lactose-free milk, or to 1–2% to yield lactose-reduced milk, meeting the dietary needs of lactose-intolerant individuals [3]. Moreover, β-gal treatment enhances the natural sweetness of milk without adding external sweeteners, because the released glucose and galactose have a higher relative sweetness than lactose [4]. This enzymatic hydrolysis also improves the solubility and mouthfeel of concentrated or frozen dairy products by preventing lactose crystallization. This property is particularly beneficial in the production of ice cream, sweetened condensed milk, and whey-derived ingredients [3].

As one of the most promising glycosidases, β-gal exhibits potent hydrolytic and transglycosylation activities. This dual activity enables it to catalyze the transfer of a galactosyl moiety to various acceptors, including lactose [5]. The synthesis of prebiotic galacto-oligosaccharides (GOS) represents the most mature application of β-galactosidase’s transglycosylation activity. This process capitalizes on the dual function of lactose, which acts both as a glycosyl donor and as an acceptor. Upon the addition of the enzyme under controlled conditions, a cascade of transgalactosylation reactions yields a diverse mixture of GOS, with the final composition depending on factors such as the degree of polymerization (DP) and the type of glycosidic bonds formed [6]. This biosynthetic route represents the most established method for industrial GOS production.

In addition to synthesizing GOS, the transgalactosylation activity of β-gal also produces various galactose-containing oligosaccharides and other galactoside compounds, exhibiting broad substrate specificity [7]. β-Gal interacts with its substrates via two distinct binding modes. The first is a deep binding mode at the catalytic site, characterized by strong interactions (non-covalent interactions, including hydrogen bonding, hydrophobic/aromatic stacking, van der Waals, and electrostatic) and high specificity. In this mode, the glycosidic oxygen is protonated as it is positioned in proximity to the acid catalyst, while the anomeric carbon of the galactosyl moiety is suitably aligned for nucleophilic attack. In contrast, the second is a shallow binding mode that involves weaker interactions and broader specificity, thereby facilitating binding of structurally diverse substrates [8,9]. As illustrated in Fig. 1a, the deep binding mode engages the –1 and +1 subsites, which are where β-gal catalyzes lactose hydrolysis. In this configuration, the β-D-galactosyl moiety is stabilized at the –1 subsite, while the glucosyl group occupies the +1 subsite. Alternatively, the shallow binding mode involves the +2 and higher subsites. This mode facilitates the transgalactosylation reactions, enabling both the synthesis of GOS and the galactosylation of other glycosyl acceptors [[10], [11], [12], [13]]. Our previous study explored the catalytic characteristics of β-gal from *Lactobacillus delbrueckii* subsp. *bulgaricus* (LDBUBgal) [8]. As illustrated in Fig. 1b, the galactosyl moiety at the –1 subsite acts as the sugar donor, engaging in hydrogen-bonding interactions with two glutamic acid residues at the active site. The sugars at the +1 and +2 subsites are the acceptors [8].

**Fig. 1:**
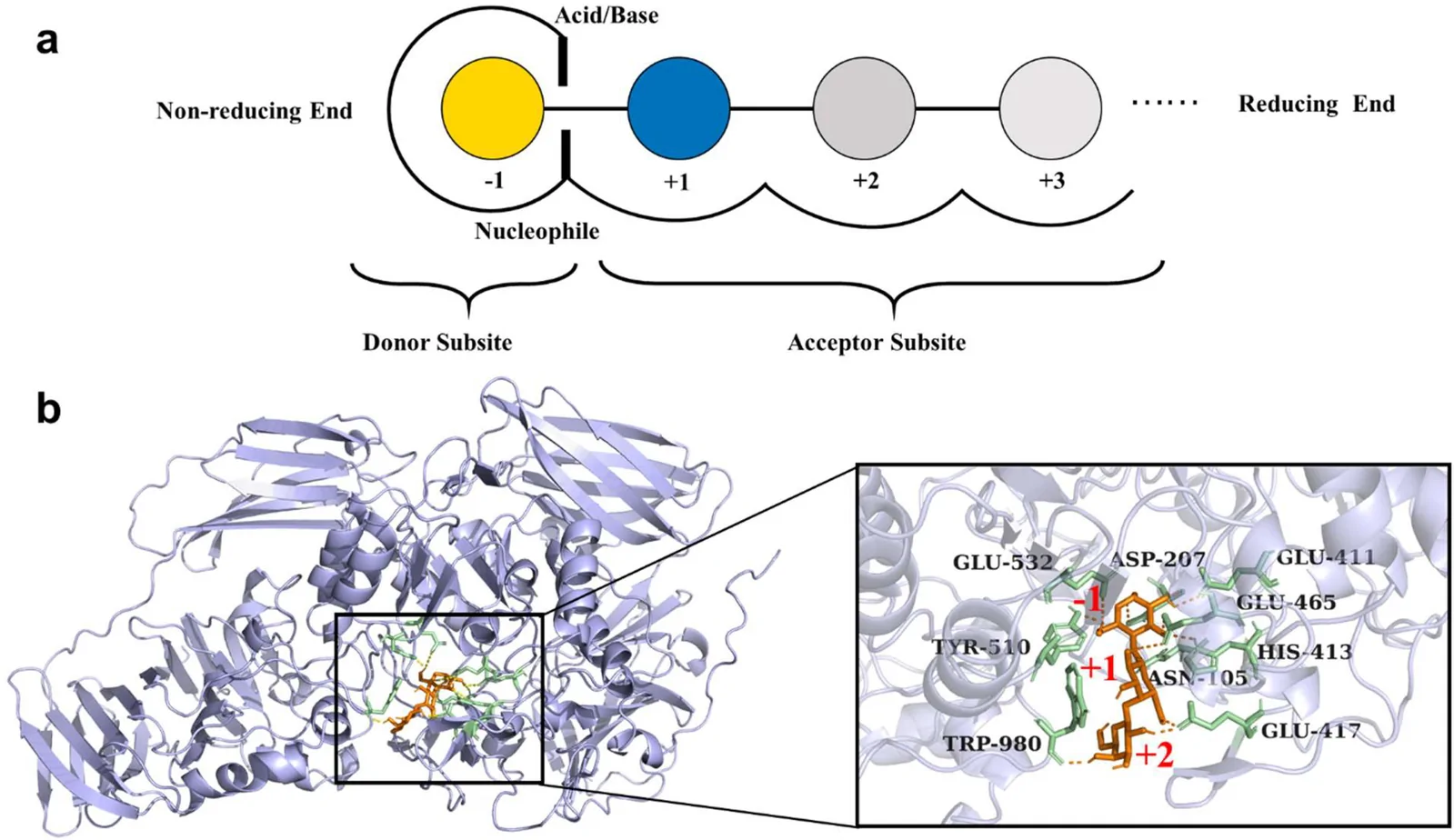
The description of the catalytic subsites of β-gal. **a**: The schematic diagram of the catalytic subsites of β-gal [8]. **b**: Molecular docking simulation of 4′-*O*-β-galactosyllactose binding to LDBUBgal [8].

β-gal can also perform glycosylation reactions on simple carbohydrate acceptors other than lactose and oligosaccharides. For instance, β-gal can catalyze the synthesis of lactulose [14] and fructosyl-galacto-oligosaccharides (FGOS) [15] from lactose and fructose. Lactulose is valued both as a prebiotic supplement and a long-established pharmaceutical for treating chronic constipation and hepatic encephalopathy [16]. β-Gal can also catalyze the synthesis of certain oligosaccharide units, such as the transfer of galactose to terminal residues of certain N-glycan chains [17].

Besides carbohydrate glycosyl acceptors, β-gal can also glycosylate certain non-carbohydrate glycosyl acceptors, including aliphatic alcohols, antibiotics, nucleosides, sugar alcohols, natural products, and amino acids [2,9]. Studies have shown that β-gal can effectively glycosylate antibiotics and natural products; moreover, glycosylation can improve the properties of these compounds; for example, glycosylated antibiotic prodrugs exhibit significantly reduced toxicity and cause less damage to organisms [9,18]. Additionally, the water solubility of certain natural products has been greatly improved by glycosylation, and their application scope has also widely expanded [19,20].

As a glycoside hydrolase with superior transglycosylation activity, β-gal possesses broad substrate specificity, enableing it to recognize different types of glycosyl acceptors to synthesize different galactosyl compounds. This article reviews the transgalactosylation of various glycosyl acceptors catalyzed by β-gal and summarizes the properties of the resulting glycosylated products.

## 2 Classification of β-gal with transglycosylation activity

β-gal is ubiquitous in nature and is found in animals, plants, and microorganisms. According to the CAZy database (http://www.cazy.org/), β-gal belongs to the glycosyl hydrolase (GH) family, which includes GH1, GH2, GH35, GH42, GH59, and GH147, with most belonging to GH2, GH35, and GH42. Most enzymes with transglycosylation activities are derived from the GH2 and GH42 families, while a few are derived from GH35.

As shown in the evolutionary tree in Fig. 2, the GH2 family, which was mainly derived from bacteria and yeast, contains the most β-gal species. For instance, the best-known β-gal is from *Escherichia coli*, and many studies have elucidated the crystal structures of the wild-type and mutant enzymes [21]. Additionally, commercially available β-gals have been isolated from *Kluyveromyces, Lactobacillus, Bifidobacterium*, and other important microorganisms. We previously investigated the catalytic properties of LDBUBgal, a classic member of the GH2 family, which has a molecular weight of 114.5 kDa and is predominantly a natural tetramer (Fig. 3a). The monomeric structure of this enzyme is shown in Fig. 3b. The galactose molecule is located deep within the substrate-binding pocket, occupying the –1 subsite, where it forms hydrogen-bonding interactions with the catalytic residues Glu465 and Glu532.

**Fig. 2:**
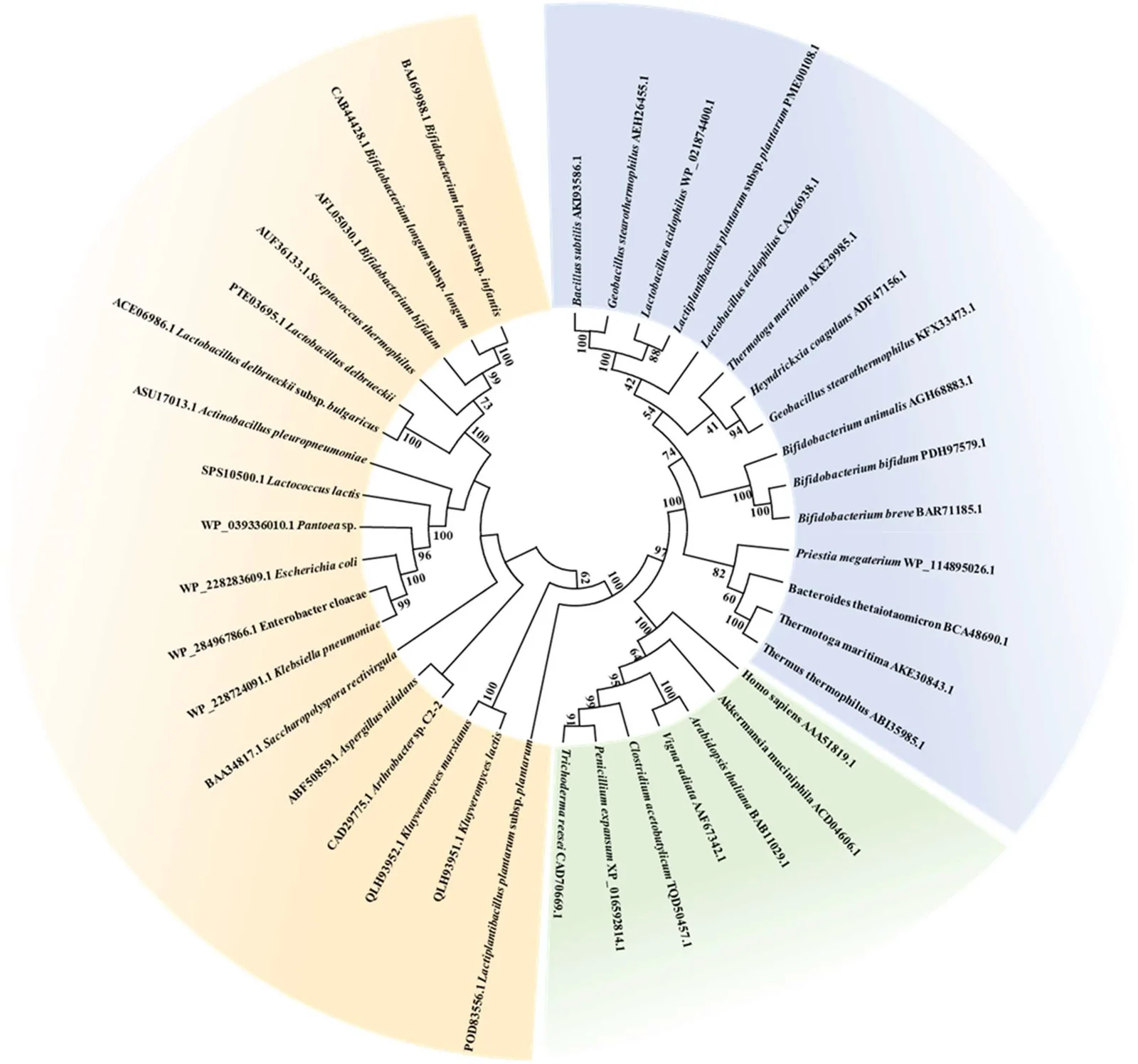
Family classification of β-gal with transglycosylation activity. The yellow, blue, and green parts represent the GH2, GH42, and GH35 families, respectively.

**Fig. 3:**
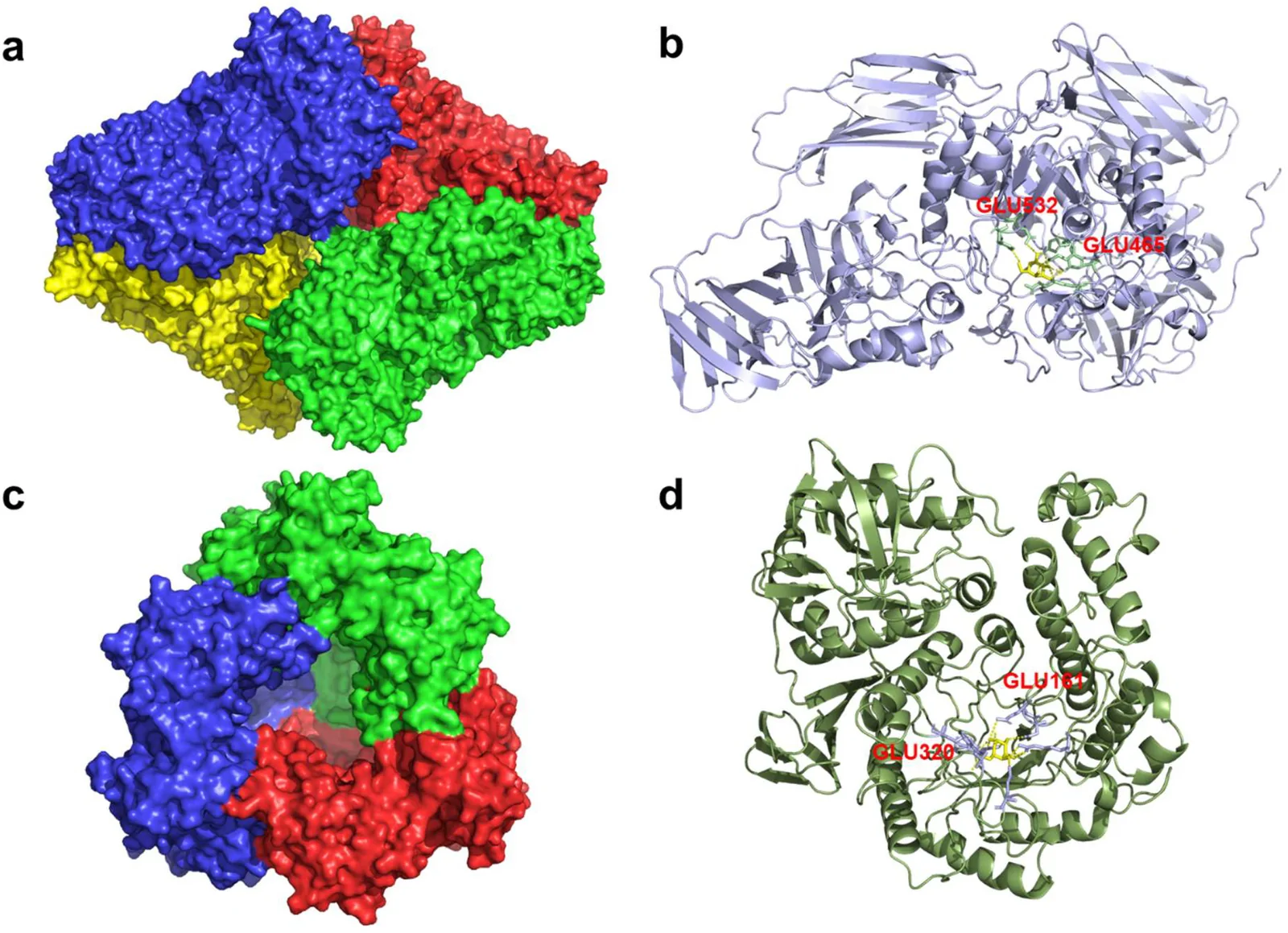
Three-dimensional structures of GH2 and GH42 β-gals. **a**: Surface presentation of tetramer with monomers in different colors of LDBUBgal. **b**: Cartoon presentation of monomer of LDBUBgal. The yellow sticks represent galactose, and the green sticks represent residues that form hydrogen bond interactions with galactose. **c**: Surface presentation of tetramer with monomers in different colors of BBBgal. **d**: Cartoon presentation of monomer of BBBgal. The yellow sticks represent galactose, and the lavender sticks represent residues that form hydrogen bond interactions with galactose.

In contrast, a GH42 β-gal from *Bifidobacterium bifidum* NCIMB 41171 (BbgalⅡ) naturally exists as a trimer (Fig. 3c), with a monomer molecular weight of 85.58 kDa, which is generally lower than that of GH2 family enzymes. As shown in Fig. 3d, a galactose molecule also binds deep within the catalytic pocket at the –1 subsite and establishes hydrogen-bonding interactions with the catalytic residues Glu161 and Glu320. In addition, the GH42 family mainly includes β-gals derived from microorganisms that can adapt to extreme environments, such as thermophilic microorganisms. These enzymes are particularly critical for the growth of microorganisms that use galactose-related polysaccharides in plant biomass as carbon sources [2]. For example, a β-gal from *Geobacillus stearothermophilus* T-6, which has significant hydrolytic activity on galactobiose and higher-molecular-weight galactose oligomers, cannot hydrolyze lactose [22].

β-gals of the GH35 family have been identified in a wide range of sources, including bacteria, fungi, plants, and animals. A distinctive β-gal from *Trichoderma reesei* exhibits broad substrate specificity. It hydrolyzes a range of disaccharides and monosaccharide galactosides, including lactose, lactulose, galactobiose, and various aryl/alkyl-β-D-galactosides. Furthermore, it is also capable of cleaving β-(1→3)- and β-(1→4)-linked galactose polymers [23]. Moreover, a bovine testis-derived β-gal catalyzes the glycosylation reaction of sucrose, isomalt, and raffinose, generating a series of novel β-(1→3)-glycosidic bond-linked GOS derivatives [24].

## 3 Glycosylation of carbohydrate acceptors by β-gal

### 3.1 Synthesis of lactose-derived GOS

#### 3.1.1 The characteristics and research status of GOS

GOS has emerged as one of the most prominent prebiotics, attracting significant research and industrial interest. It can regulate the intestinal flora, increase the abundance and metabolic activity of probiotics, reduce the adhesion of harmful bacteria, regulate the gut immune system, help manage metabolic diseases, and remain stable over a wide range of pH values and temperatures [8,25]. The molecular chain of GOS contains 2 to 8 monosaccharides, of which one end is a glucose molecule, and the rest are galactose molecules. Disaccharides composed of two galactose moieties linked through transglycosylation are also considered GOS [26]. As classic prebiotics, GOS transits through the upper gastrointestinal tract undigested, reaching the colon, where they exert their beneficial effects by selectively promoting the growth of probiotics such as *Bifidobacterium* and *Lactobacillus* species while inhibiting the growth of pathogenic enterobacteria [27]. This leads to favorable modulation of gut microbiota composition, which positively influences immune responses and markers of metabolic syndromes [[28], [29], [30]]. Furthermore, microbial fermentation of GOS in the colon produces short-chain fatty acids, including acetate, propionate, butyrate, and lactate. These serve as energy sources for colonic epithelial cells, lower the intestinal pH, facilitate the absorption of calcium and magnesium ions, and contribute to the reduction of pro-inflammatory markers [[31], [32], [33]].

With the continuous improvement of living standards, people’s intake of meat and dairy products has been increasing. This change in dietary structure has directly led to a surge in hypertension, diabetes, and various oral and digestive system diseases; therefore, the demand for functional food has also been increasing. As a classic prebiotic health food, GOS has long been popular with consumers for its excellent physiological effects. Market research estimated the global GOS market in 2025 to be between $0.92 billion and $1.16 billion, with strong growth projected through the next decade driven by increasing demand for prebiotics in functional foods and infant formula [9,34]. The industrial production of GOS originated in Japan, and commercial production of GOS began in Europe and the United States. Nowadays, many Chinese companies are also targeting the GOS industry. However, two major problems remain in the industrial production of GOS. First is the difficulty in the separation and purification of GOS; at present, the mass fraction of many GOS products in the China's domestic market is <57%. Second, the transglycosylation activity of existing commercial β-galactosidases still needs improvement [35], with current GOS yields remaining below 50%. Therefore, discovering new β-gals with excellent performance remains a key focus of future research on the industrialization of GOS, with considerable research value and development prospects. The main manufacturers of GOS commercial production include Yakult Co., Ltd. (Japan), WELLNEO SUGAR Co., Ltd. (Japan), Corn Products International Inc. (USA), FrieslandCampina Domo B.V. (Netherlands), Clasado Ltd. (UK), Baolingbao Biology (China), and Quantum Hi-Tech Biotechnology Co., Ltd. (China) [36]. A summary of the product information for some commercialized GOS products is shown in Table 1.

**Table 1:** Summary of some commercialized GOS products.

GOS product	Manufacturer	Enzyme source	GOS mass fraction (%)
Oligaomat 55	Japan Yakult Co., Ltd.	*Aspergillus oryzae*	50∼60
TOS-100	Japan Yakult Co., Ltd.	*Streptococcus thermophilus*	99∼100
CUP-oligo	Tokyo Nissin Sugar Co., Ltd.	*Cryptococcus laurentli*	70
Purimune powder	Illinois Corn Products International Co., Ltd.	*Bacillus circulans*	90∼92
Vivinal	FrieslandCampina Domo B.V.	*Bacillus circulans*	57∼59
Bimuno Syrup and powder	Claxado LTD., Milton Keynes, UK	*Bifidobacterium bifidum*	48∼55
Bowling Bao GOS powder	China Bowling Treasure Biology Co., Ltd.	——	57∼90
Quantum High-tech brand GOS	China Quantum High-tech Biology Co., Ltd.	——	30∼90

#### 3.1.2 GOS synthesis by β-gal

The synthesis of GOS generally uses lactose as a substrate; the transglycosylation reaction by β-gal generates complex GOS mixtures with varying DPs [37]. The basic reaction mechanism, in which β-gal follows a double-displacement pathway, is shown in Fig. 4a. First, β-gal combines with a lactose molecule to form an intermediate, and then removes a glucose molecule to generate a β-gal-galactosyl intermediate. When the lactose concentration in the system is low, the water molecule preferentially acts as a glycosyl acceptor; the galactosyl and the water hydroxyl groups then combine to form galactose. In contrast, when the lactose concentration in the system is high, it promotes lactose as a glycosyl acceptor. The galactosyl moiety then combines with the hydroxyl group of lactose to form a trisaccharide, which can subsequently act as a glycosyl acceptor to generate a tetrasaccharide and higher-degree GOS, resulting in a series of GOS with varying DPs [1]. β-gals from different sources catalyze the production of GOS with varying DPs and glycosidic linkages. Fig. 4b illustrates the structural formulas of galactobiose containing different glycosidic bonds, as well as trisaccharides and tetrasaccharides linked by β-(1→4)-glycosidic bonds.

**Fig. 4:**
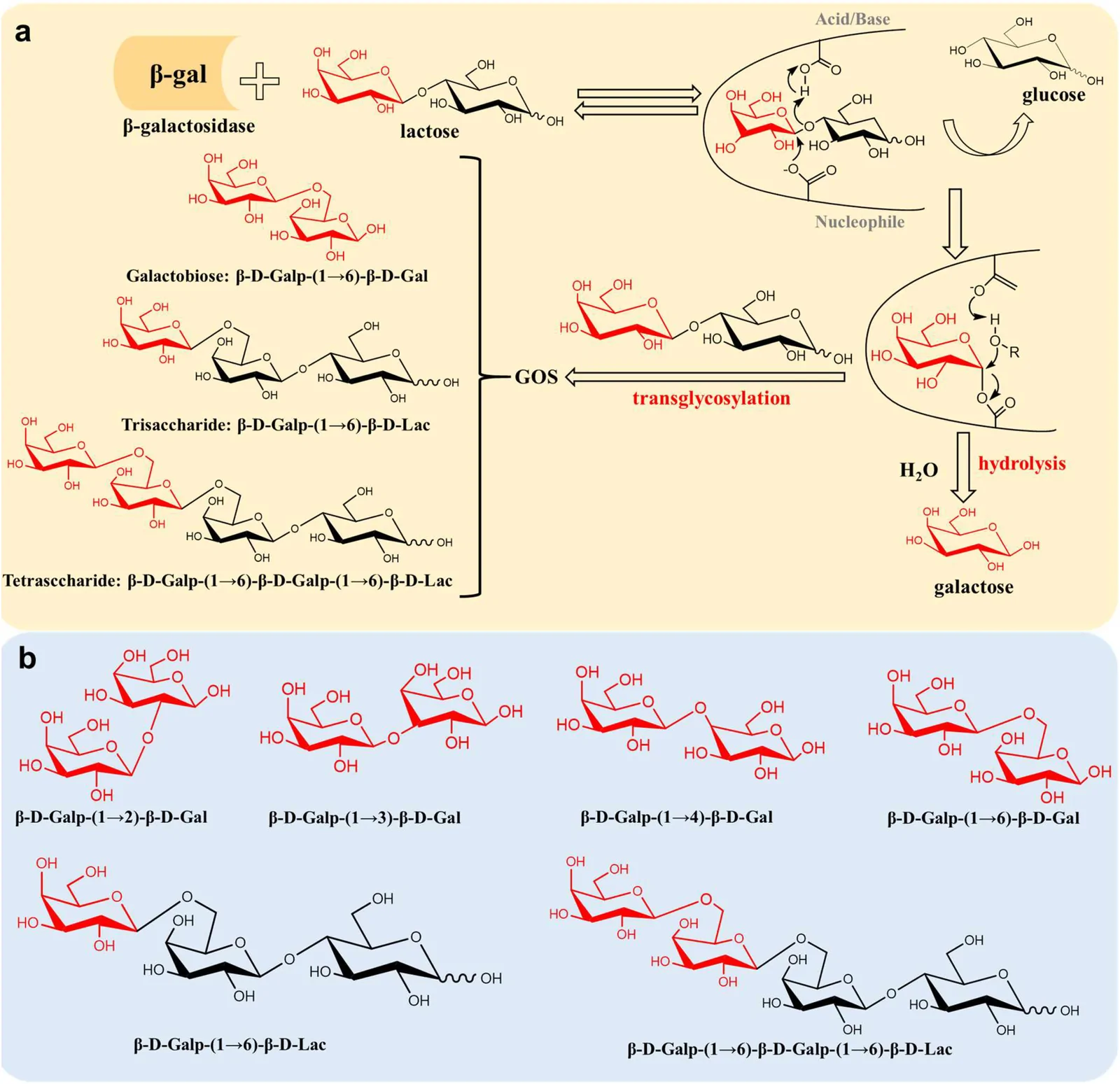
Schematic diagram illustrating the β-gal-catalyzed hydrolysis and transglycosylation of lactose, leading to the production of GOS with diverse DP and glycosidic bond types. **a**: Shown in the figure β-gal follows a double displacement mechanism, first forming glycosylase intermediates, and then hydrolyzing or transglycosylation with water or sugar as receptors. The glycosidic bonds of the transformed GOS are all β-(1→6)- glycosidic bonds, and the galactose and galactosyl are shown in red. **b**: Galactobiose with different glycosidic bonds, trisaccharides, and tetrasaccharides. In which all galactosyl moieties are shown to be linked via β-(1→6) glycosidic bonds.

Generally, β-galactosidases derived from microorganisms exhibit higher productivity than those derived from plants and animals and can significantly reduce production costs [1]. Most β-gals used in the synthesis of GOS, whose sources include *Aspergillus, Kluyveromyces, Bacillus, Lactobacillus*, and *Bifidobacterium*, belong to the GH2 family [38]. Among the various microbial β-gals, those produced by *Aspergillus, Kluyveromyces*, and *Bacillus* are the most widely used for commercial GOS production [39]. For example, a commercial β-gal (Lactazyme-B) derived from *Bacillus circulans* can achieve a maximum GOS yield of 63.4% [40]. Table 2 summarizes the reported cases of GOS synthesis catalyzed by β-gals derived from the above three microorganisms.

**Table 2:** Synthesis of GOS by β-gals from *Aspergillus, Kluyveromyces*, and *Bacillus.*

β-gal source	Catalyst	Temperature (°C)	pH	Initial lactose concentration (% w/w)	Yield (%)	DP	Glycosidic bond	Reference
*Aspergillus oryzae*	Purified enzyme	55	4.5	19	24.3	2∼5	——	[108]
*Aspergillus oryzae*	Purified enzyme	40	4.5	40	21	2∼3	——	[42]
*Aspergillus oryzae*	Purified enzyme	40	4.5	42	26	2∼3	β-(1→6)-	[109]
*Aspergillus oryzae*	Purified enzyme	40	4.6	50	16	—	——	[110]
*Aspergillus oryzae*	Purified enzyme	40	6.7	4.5	9.8	2∼3	β-(1→6)-	[111]
*Aspergillus oryzae*	Purified enzyme	47.5	4.5	50	29	—	——	[112]
*Kluyveromyces lactis*	Purified enzyme	45	6.5	19	32	2∼4	——	[108]
*Kluyveromyces lactis*	Purified enzyme	40	7.5	40	11	2∼4	β-(1→6)-	[42]
*Kluyveromyces lactis*	whole cell	35	8.0	30	35	—	——	[41]
*Kluyveromyces lactis*	Purified enzyme	40	6.7	4.5	15	2∼3	β-(1→6)-	[111]
*Bacillus circulans*	Purified enzyme	40	7	40	41	2∼5	β-(1→4)-	[42]
*Bacillus circulans*	Purified enzyme	40	6.7	4.5	16	2∼4	β-(1→4)-	[111]
*Bacillus circulans*	Immobilized pure enzyme	60	6	50	39	2∼6	——	[113]

Studies have used ethanol to treat *Kluyveromyces lactis* cells, thereby increasing their permeability and facilitating substrate entry and product release. These treated cells are then incubated with lactose to synthesize GOS. The innovation of this research is that the ethanol produced by this process can be reused to initially permeabilize cells. After supercritical fluid extraction and nanofiltration, the purity of the final GOS can reach 95% [41]. Another study explored the ability of β-gals derived from *Bacillus circulans, Aspergillus oryzae*, and *Kluyveromyces lactis* to synthesize GOS. The results showed that *Bacillus circulans* β-gal produced the highest GOS yield (41%), followed by *Aspergillus oryzae* β-gal (21%) [42]. The composition and structure of the resulting GOS were further analyzed. As summarized in Table 2, β-gals derived from *Aspergillus oryzae* and *Kluyveromyces lactis* mostly catalyzed the synthesis of β-(1→6)- glycosidic bonds, while *Bacillus circulans* β-gal catalyzed the formation of β-(1→4)-glycosidic bonds [42]. Some studies used a combination of two β-gals derived from *Aspergillus oryzae* and *Kluyveromyces lactis* to catalyze the synthesis of GOS. The combined application of the two enzymes increased the yield of GOS with a higher DP from 38.5% to 40.0%. This improvement was primarily attributed to the enhanced production of longer-chain oligosaccharides: the yield of tetrasaccharides increased from 6.7% to 12.8%, while that of pentasaccharides rose from 0.4% to 3.3% [43]. Furthermore, the structural diversity of the final GOS products may be enhanced owing to the distinct glycosidic linkage preferences of the two β-gals [43]. The above representation of GOS production uses the ratio of GOS mass concentration (g/L) to initial lactose mass concentration (g/L), i.e., the GOS yield is expressed as a percentage of the initial lactose concentration [31].

In addition to those from *Aspergillus, Kluyveromyces*, and *Bacillus*, β-gals derived from *Bifidobacterium* and *Lactobacillus* also have significant value in GOS production, attracting considerable research attention. *Bifidobacterium* and *Lactobacillus* species offer an additional advantage beyond their “Generally Recognized as Safe” (GRAS) status and long-standing use in food fermentation: GOS synthesized using β-gals derived from these genera are most likely to selectively promote their proliferation and metabolic activity in the gut, thereby amplifying their probiotic efficacy [44]. *Bifidobacterium* species capable of producing β-gal with transglycosylation activity include *B. bifidum* [[44], [45], [46]], *B. infantis* [47,48], *B. breve* [49], *B. angulatum, B. adolescentis*, and *B. pseudolongum* [1]. *Lactobacillus* species capable of producing β-gal with transglycosylation activity include L. *plantarum* [[50], [51], [52]], L. *pentosus* [37,53], *Lactobacillus brevis* [54], *Lactobacillus acidophilus* [55], L. *fermentum* [56], L. *sakei* [57], L. *delbrueckii* subsp. *bulgaricus* (*Lactobacillus bulgaricus*) [8,58,59], L. *reuteri* [60], and L. *paracasei* [61].

Most *Lactobacillus* species produce *Lac*LM-type β-gal, a heterodimer encoded by the two genes *Lac*L and *Lac*M are members of the GH2 family. These species include L. *pentosus* KUB-ST10– 1, *L. acidophilus* R22, L. *plantarum* WCFS1, L. *sakei* Lb790, and L. *reuteri* L103 [53]. While the *Lac*Z-type β-gal encoded by the *Lac*Z gene is uncommon in *Lactobacillus*, a few species, including *L. bulgaricus* DSM20081, produce this type of enzyme [59]. β-gals of the *Lac*Z type are more common in *Bifidobacterium*: β-gals produced by *B. bifidum* NCIMB 41,171, *B. infantis* HL96, *B. breve* DSM 20213, and *B. longum* CCRC 15708 are all of the *Lac*Z type [49,56,59]. Most *Lactobacillus*- and *Bifidobacterium*-derived β-gals with transglycosylation activity belong to the GH2 family; only a few strains belong to the GH42 family, such as BbgII [45] and the β-gal of *B. infantis* HL96 (β-gal III) [62]. β-gals of the GH2 family usually have stronger transglycosylation activity, and their transglycosylation properties have been extensively studied [49].

The source of β-gal is the most significant factor influencing GOS synthesis. Different sources lead to differences in the yield of synthesized GOS, the composition of the product, and the β-glycosidic bonds generated, including β-(1→3), β-(1→4), and β-(1→6) [27]. As shown in Fig. 4, β-gal catalyzes the generation of GOS with various DPs and glycosidic bonds, where the galactobiose (composed of two galactose molecules) formed by the combination of two galactose molecules is also considered GOS. The yields of GOS catalyzed by different *Lactobacillus*-derived and *Bifidobacterium*-derived β-gals under different reaction conditions ranged from 10% to 70%. After optimizing the reaction, the GOS yield ranged from 30% to 40%. Only a few β-gals have a GOS yield exceeding 50%. For example, the GOS yield of β-gal derived from *B. infantis* HL96 reached 63% [48], whereas that derived from *B. bifidum* NCIMB41171 exceeds 50% whether it is catalyzed by whole cells or recombinant enzymes [44]. Among *Lactobacillus* strains, the β-gal produced by the L. *bulgaricus* DSM2008147 strain has a GOS yield of approximately 50% [59]. Although the β-gal derived from *Lactobacillus* and *Bifidobacterium* exhibit considerable strain specificity, only a few can achieve GOS yields of 60% or higher, with the DP of the resulting GOS generally ranging from 2 to 5 [63]. An LDBUBgal was previously screened in our laboratory. After lactose induction, the enzyme was successfully expressed extracellularly in *E. coli* and demonstrated exceptional GOS synthesis performance, producing 460.2 g/L of GOS from 60% lactose with a 71% conversion rate; the DP of the synthesized GOS reached 6 [8]. This yield represents the highest value reported in the literature to date.

In another study, non-lactodisaccharides, trisaccharides, and tetrasaccharides in GOS synthesized by the β-gal of L. *acidophilus* R22 accounted for 15.5%, 19.0%, and 4.0% of the total sugar mass, respectively [55]. Similarly, the proportions of non-lactodisaccharides, trisaccharides, and tetrasaccharides in GOS products synthesized by the β-gal of L. *plantarum* WCFS1 were 19%, 21%, and 1.3%, respectively [64]. Another study involving β-gal from *B. bifidum* NCIMB 41171 synthesized a GOS mixture containing 25% disaccharides, 35% trisaccharides, 25% tetrasaccharides, and 15% pentasaccharides [46]. Naturally, the complexity of synthesizing sugar chains increases with their length. Most β-galactosidases can synthesize sugar chains of up to four monosaccharide units; enzymes that can synthesize sugar chains longer than tetrasaccharides have strong transglycosylation activity. Table 3 summarizes β-gals from different sources and their abilities to synthesize GOS.

**Table 3:** Synthesis of GOS by β-gals derived from *Lactobacillus* and *Bifidobacterium.*

β-gal source	Catalyst form	pH	Temperature (°C)	Initial lactose concentration (%, w/v)	Yield (%)	DP	Glycosidic bond	Reference
*Bifidobacterium longum* RD47	Crude enzyme	8.5	45	36% (w/v)	25.3	2∼4	β-(1→4)-	[31]
*Bifidobacterium breve* DSM 20213	Purified enzyme	7.0	50	20%	33	2∼3	β-(1→6)- β-(1→3)-	[49]
*Bifidobacterium breve* DSM 20213	Purified enzyme	6.5	55	20%	44	2∼3	β-(1→6)- β-(1→3)-	[49]
*Bifidobacterium breve* B24	Purified enzyme	7.0	45	34%	42	—	——	[114]
*Bifidobacterium bifidum* NCIMB41171	Crude enzyme	6.8	65	43% (w/w)	54.8	2∼3	——	[44]
*Bifidobacterium bifidum* NCIMB41171	Whole cell	6.8	65	43% (w/w)	52.2	2∼3	——	[44]
*Bifidobacterium bifidum* NCIMB41171	Purified enzyme	6.0–6.6	52–56	——	52.3	2∼4	——	[38]
*Bifidobacterium bifidum* NCIMB41171	Whole cell	6.8–7.0	40	55% (w/w)	35%	2∼5	——	[46]
*Bifidobacterium bifidum* BB-12	Crude enzyme	7.5	55	30% (w/w)	37.6	——	β-(1→4)- β-(1→6)- β-(1→3)- β-(1→2)-	[115]
*Bifidobacterium bifidum*	Crude enzyme	6.0	37	20%	44	——	——	[116]
*Bifidobacterium infantis* DSM-20088	Crude enzyme	7.5	55	30% (w/w)	47.6	——	β-(1→4)- β-(1→2)- β-(1→3)- β-(1→6)-	[115]
*Bifidobacterium adolescentis* ANB-7	Crude enzyme	7.5	55	30% (w/w)	43.1	——	β-(1→4)- β-(1→6)-	[115]
*Bifidobacterium angulatum*	Crude enzyme	7.5	55	30% (w/w)	43.8	——	β-(1→4)- β-(1→3)- β-(1→6)-	[115]
*Bifidobacterium pseudolongum* DSM-20099	Crude enzyme	7.5	55	30% (w/w)	26.8	——	β-(1→4)- β-(1→3)- β-(1→2)- β-(1→6)-	[115]
*Bifidobacterium* infantis HL96	Crude enzyme	7.0	45	36%	40.6	2∼4	——	[47]
*Bifidobacterium infantis* HL96	Purified enzyme	7.5	60	30%	63	——	——	[48]
*Bifidobacterium logum* BCRC 15,708	Crude enzyme	6.8	45	40%	32.5	2∼4	——	[117]
*streptococcus thermophilus* DSM20259	Crude enzyme	6.5	37	20%	50	2∼3	β-(1→6)- β-(1→3)-	[118]
*streptococcus thermophilus* ST61	Crude enzyme	6.8	40	30%	53.45 g/L	——	——	[119]
*Lactobacillus delbrueckii* subsp. *bulgaricus*	Crude enzyme	6.5	30/37	60%	460.2 g/L	2∼6	β-(1→6)-	[8]
*Lactobacillus pentosus* KUB-ST10–1	Purified enzyme	6.5	30	20%	31	2∼3	β-(1→6)- β-(1→3)-	[53]
*Lactobacillus plantarum* MCC2156	Permeabilized cell	7.0	50	40% (w/v)	34%	2∼4	——	[51]
*Lactobacillus plantarum* 70,810	Whole cell	7.0	45	40%	44.3	2∼3	——	[52]
*L. brevis* PLA28	Purified enzyme	6.5	30	20%	——	——	——	[54]
*L. bulgaricus* DSM20081	Purified enzyme	6.5	30	20%	49.5	2∼3	β-(1→6)- β-(1→3)-	[59]
*L. bulgaricus L3*	Immobilized enzyme	7.6	45	40%	49	——	——	[120]
*Lactobacillus fermentum* K4	Purified enzyme	6.5	45	40% (w/v)	37	——	——	[56]
*Lactobacillus sakei* Lb790	Purified enzyme	6.5	37	21%	41	2∼3	β-(1→6)- β-(1→3)-	[57]
*L. acidophilus* R22	Purified enzyme	6.5	30	20%	38.5	2∼3	β-(1→6)- β-(1→3)-	[55]
*Lactobacillus reuteri* L103	Purified enzyme	6.5	30	20%	38	2∼3	β-(1→6)- β-(1→3)-	[60]
*Lactobacillus paracasei* YSM0308	Crude enzyme	6.5–7.0	30	40%	19.4	2∼4	——	[61]
*Lactobacillus plantarum* WCFS1	Purified enzyme	6.5	37	20%	41	2∼3	β-(1→6)- β-(1→3)-	[64]
*Lactobacillus pentosus* BFP32	Crude enzyme	7.0	50	60% (w/w)	13.9	2∼5	——	[50]
*Lactobacillus plantarum* MCC2156	Permeabilized cell	7.0	50	40%	34	2∼5	——	[121]

Commercial GOS is generally dominated by β-(1→4)-glycosidic bonds, which is somewhat different from the linkage profile of β-gals derived from *Lactobacillus* and *Bifidobacterium*. β-gals from *Lactobacillus* tend to synthesize β-(1→6)- and β-(1→3)- glycosidic bonds, as seen for the β-gal from *L. bulgaricus* DSM20081 [59]. *L. reuteri* L103 [60], *L. plantarum* WCFS1 [64], and *L. sakei* Lb790 [57] are particularly prone to synthesizing β-(1→6)-glycosidic bonds. β-gals derived from *Bifidobacterium* have similar properties. Most of the disaccharides and trisaccharides synthesized by β-gal derived from *B. breve* DSM 20213 contain β-(1→6)- and β-(1→3)-glycosidic bonds, where only a few trisaccharides are linked by β-(1→3)-glycosidic bonds [65].

In addition, some β-gals from the GH42 family exhibit a preference for forming β-(1→6)- and β-(1→3)-glycosidic linkages. For instance, two *Pantoea anthophila* strains, Bac 55.2 and Bac 69.1, isolated from the traditional Mexican beverage “tejuino,” efficiently synthesize GOS under optimized conditions (400 g/L lactose, 50 °C, 15 U/mL β-gal activity using ethanol-permeabilized cells) [66]. Their GOS yields reached 136 g/L and 145 g/L, respectively, with lactose conversions exceeding 94%. These strains predominantly produced di- and trisaccharides containing β-(1→3) and β-(1→6) linkages, demonstrating high transgalactosylation activity [66]. Subsequently, a β-gal derived from *P. anthophila* (B-gal42) was heterologously expressed in *E. coli* and used to synthesize GOS from lactose or milk whey. This enzyme exhibited high stability and, consistent with the catalytic behavior of the wild-type strains, mainly formed GOS with β-(1→6)- and β-(1→3)- glycosidic linkages [67]. The GOS yield reached 40% (w/w) at a lactose concentration of 400 g/L, corresponding to 86% lactose conversion. Both milk whey and pure lactose resulted in a GOS yield of 38% under conditions with 300 g/L substrate, with identical product profiles [67]. These results indicate that B-gal42 can efficiently produce high-value GOS from low-cost whey, showing great potential for industrial applications.

However, β-gals derived from *Bifidobacterium* also catalyze the production of GOS linked by β-(1→4)-glycosidic bonds. For instance, β-(1→4)-glycosidic bonds exist in both trisaccharides and tetrasaccharides synthesized by β-gal from the *B. longum* RD47 strain [31]. As summarized in Table 3, compared with those from *Lactobacillus*, the β-gals from *Bifidobacterium* can generate more types of glycosidic bonds. Hence, *Bifidobacterium* species are a source of β-gal with great potential to catalyze galactosylation reactions to produce diverse products.

#### 3.1.3 Advances in the enzyme engineering of β-gal

Enzyme engineering to enhance the transgalactosylation activity of β-gals is relatively well established. Through techniques such as site-directed mutagenesis and directed evolution, the transgalactosylation/hydrolysis ratio of β-gals can be effectively increased, thereby improving the efficiency of GOS synthesis and enabling precise control over the structures of the resulting products [9]. A recent study resolved the cryo-electron microscopy structure of a β-gal derived from *Paenibacillus barengoltzii*, revealing an expanded acidic catalytic pocket. Distal mutations in this enzyme that enhance GOS synthesis were identified using structure-guided strategies and the machine learning platform MECE. The best-performing mutant, H331V, achieved a GOS yield of 76.57% using 35% (w/v) lactose as the substrate. Molecular dynamics simulation results further indicated that the distal mutation increased the rigidity of the loops surrounding the catalytic pocket [68]. Although mutations at the –1 subsite of most β-gals can enhance GOS synthesis capacity, they often lead to a substantial decrease in overall activity (<10%). In one study, three commercial β-gals were engineered to develop a fine-tuning strategy involving aromatic residue exchanges and substitutions among threonine, serine, and alanine residues. This approach yielded mutants with residual activities ranging from 30% to 357%. Compared with the wild-type enzyme, these mutants achieved 1.2–18% higher GOS yields and produced 2.9–11.8 g/L more GOS from skim milk, with a lactose conversion rate of 90%. This strategy can also be extended to other glycoside hydrolases [69]. Furthermore, Xiang et al. [70] performed a semi-rational design on a β-gal from *Aspergillus oryzae* [70]. By disrupting hydrogen bonds, altering hydrophobicity, and expanding the catalytic pocket, they obtained a mutant, M3, which achieved an activity of 228.2 U/mL when expressed in *Aspergillus niger* and a GOS yield of 62.3%,. Based on these results, they proposed a “non-polar amino acid scanning” strategy. When applied to three β-galactosidases, this strategy increased GOS production by 30–40% in two cases. This study provided excellent catalysts for GOS production and a rapid engineering strategy for β-gal modification.

In another study, a cold-active β-gal from the GH42 family of the Antarctic haloarchaeon *Halorubrum lacusprofundi* was engineered, and double mutants were constructed to analyze their temperature-dependent effects. The double mutations included tandem residue pairs (N180T/A181T and T383A/S384A) and pairwise combinations of single-point mutations (N251D, F387L, I476V, and V482L). The results showed that all double mutants exhibited either higher activity than the wild-type enzyme at moderate temperatures or lower activity at colder temperatures. The tandem mutations affected both the *K_m_* and *k_cat_*. Among the distal combination mutants, pairing with F387L (located 19 Å from the active site) affected the *K_m_*, while pairing with I476V (located 25 Å from the active site) affected the *k_cat_*. This study demonstrated that combinations of distal residues finely regulate temperature-dependent catalysis through alterations in the hydration shell and/or perturbations in internal packing [71].

### 3.2 Glycosylation of other simple carbohydrates by β-gal

β-gal can glycosylate carbohydrates other than lactose, such as fructose and sorbose. β-Gal-catalyzed synthesis from lactose and fructose is a major method for lactulose production. Kim et al. [14] cloned and expressed a thermostable β-gal from *Sulfolobus solfataricus* in *E. coli*, and used the recombinant enzyme to catalyze the synthesis of lactulose from lactose and fructose [14]. The optimal substrates for the reaction were 40% (w/v) lactose and 20% (w/v) fructose, and the optimal enzyme concentration was 3.0 U/mL. Under the above reaction conditions, β-gal produced approximately 50 g/L of lactulose, with a productivity of approximately 8.3 g/L/h. Lee et al. [72] used *Kluyveromyces lactis*-derived β-gal to convert lactose and fructose to lactulose, and used 50% ethanol to permeabilize the cells. Following optimization, the permeabilized cells demonstrated enhanced performance, converting approximately 20 g/L lactose within 3 h and achieving a lactulose productivity of 6.8 g/L/h. This is the first report that used permeabilized yeast cells to synthesize lactulose.

β-gal can also produce FGOS from lactulose and lactose. One study examined the ability of β-gals derived from *Aspergillus oryzae, Kluyveromyces lactis*, and *Bacillus circulans* to catalyze the synthesis of FGOS from lactulose. Among them, the β-gal from *Aspergillus oryzae* had the strongest ability to synthesize FGOS. The reaction was optimized using response surface methodology, and the yield of FGOS reached 0.41 g/g [15]. Fig. 5a shows the structures of lactulose and FGOS with various DPs.

**Fig. 5:**
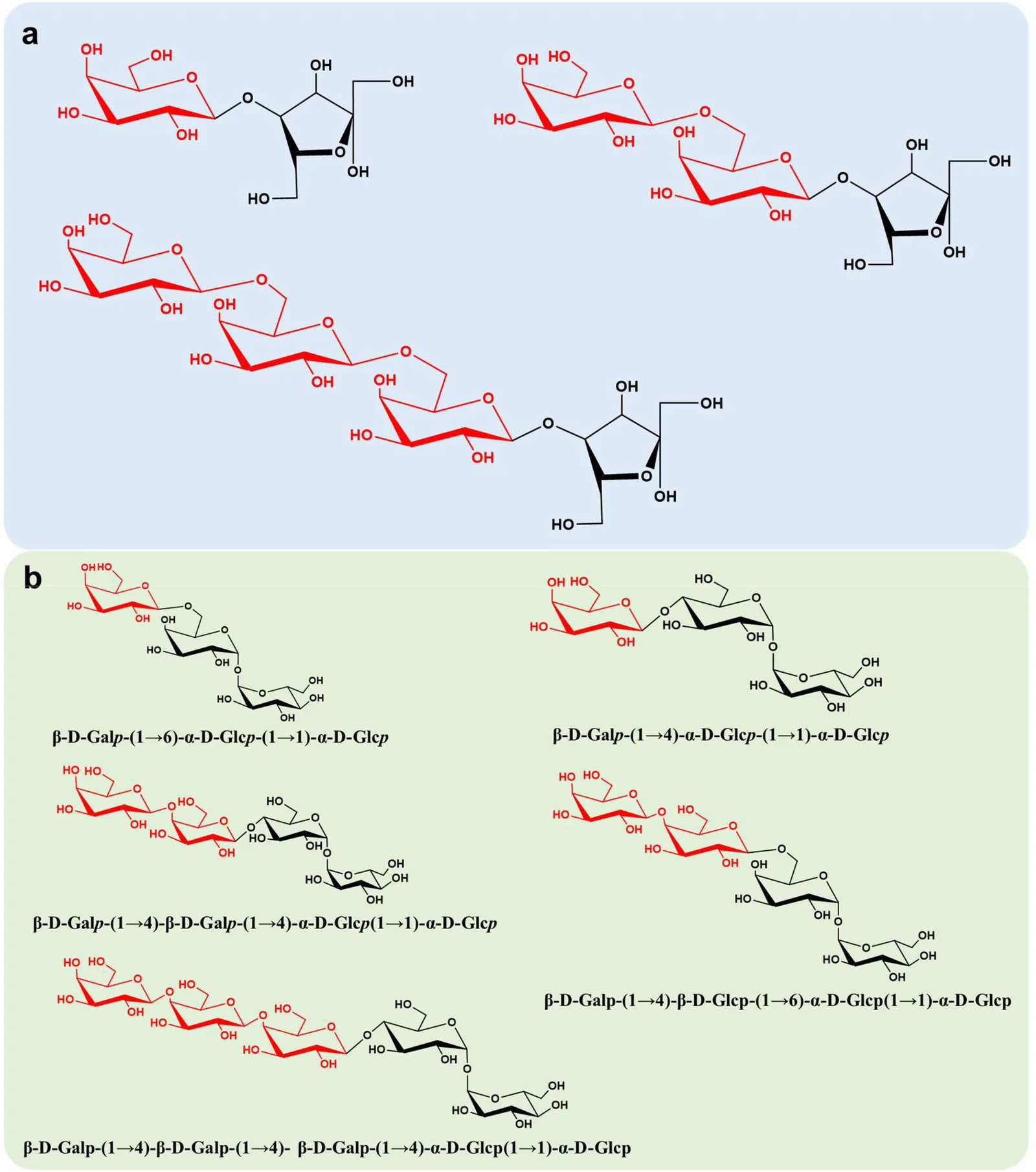
Structures of lactulose, FGOS, and TGOS. **a**: lactulose, fructosyl-GOS trisaccharide, and FGOS tetrasaccharide. **b**: Structures of TGOS containing 1∼3 galactosyl. In which all galactosyl moieties are shown to be linked via β-(1→6)- glycosidic bonds.

A series of monosaccharides and disaccharides that can be used as glycosyl acceptors includes trehalose, sucrose, mannose, isomaltose, and isomaltulose, while the trisaccharides that can be used include raffinose. One β-gal derived from *E. coli* can efficiently modify trehalose through transglycosylation to obtain several trehalose derivatives containing 1 to 3 galactosyl groups [73]. Fig. 5b shows a series of trehalose-galacto-oligosaccharides (TGOS) with various DPs.

In addition to microbial β-gals, animal-derived β-gals also exhibit strong transglycosylation activity. Schroder et al. [24] reported isolating a β-gal derived from bovine testis. In this study, lactose was used as the glycosyl donor; sucrose, isomaltulose, isomalt, and raffinose were used as the glycosyl acceptors, catalyzing the generation of a series of novel β-(1→3)-glycosidic bond-linked galactosyl derivatives, which are: β-D-Galp-(1→3)-α-D-Glcp-(1→2)-β-D-Fruf, β-D-Galp-(1→3)-α-D-Glcp-(1→6)-α/β-D-Fruf, β-D-Galp-(1→3)-α-D-Glcp-(1→6)-D-Glcp, α-D-Galp-(1→6)-(β-D-Galp-(1→3))-α-D-Glcp-(1→2)-β-D-Fruf, and β-D-Galp-(1→3)-α-D-Glcp-(1→2)-β-D-Fruf-(1→2)-β-D-Fruf. Fig. 6 shows the structures of the above novel β-(1→3)-glycosidic bond-linked galactosyl derivatives.

**Fig. 6:**
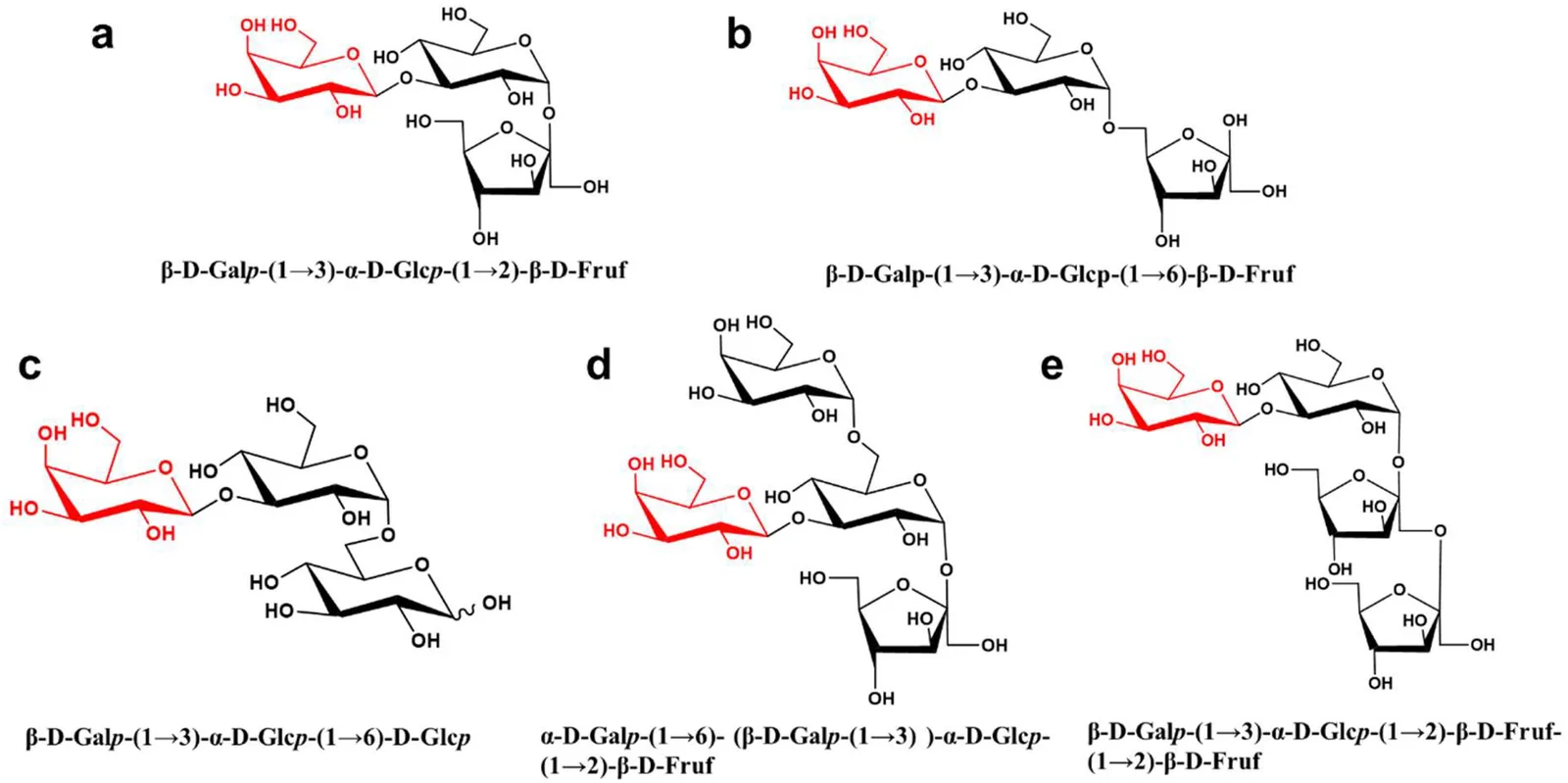
A series of novel galactosyl derivatives linked by β-(1→3)- glycosidic bonds. **a**: galactosyl sucrose. **b**: galactosyl isomaltulose. **c**: galactosyl isomalt. **d**: galactosyl raffinose. E: galactosyl 1-kestose.

### 3.3 Glycosylation of N-glycans by β-gal

β-gal also catalyzes the synthesis of galactose-containing oligosaccharides, which serve as essential building blocks for the glycan chains of glycoproteins and glycolipids. These glycans, which are rich in biological information, participate in various biological processes [7,74]. Analogous to GOS synthesis, the regioselectivity of β-gal in the glycosylation of glycan chains is largely dictated by the enzyme’s source. As early as 1996, it was reported that β-gal catalyzes the transfer of the β-D-galactosyl group from 4-nitrophenyl-β-D-galactoside (*p-*NPG) to the hydroxyl of 2-acetylamino-2-deoxygalactoside (GalNAc) and 2-acetylamino-2-deoxyglucoside (GlcNAc) [17]. This resulted in six N-glycans, including Gal-β-(1→3)-GalNAc, Gal-β-(1→4)-GalNAc, Gal-β-(1→6)-GalNAc, Gal-β-(1→3)-GlcNAc, Gal-β-(1→4)-GlcNAc, and Gal-β-(1→6)-GlcNAc [17]. Their structures are shown in Fig. 7a. This study showed that *Penicillium multicolor-*derived and *Aspergillus oryzae*-derived β-gals tend to form β-(1→6)-linked disaccharides, while β-gal derived from *Bifidobacterium bifidum* tends to form β-(1→4)- and β-(1→6)-glycosidic bonds. Gal-β-(1→3)-GlcNAc and Gal-β-(1→4)-GlcNAc dominate among the disaccharides catalyzed by β-gal derived from *Streptococcus* 6646 K [17]. These results show that the synthesis of oligosaccharide units with different glycosidic bonds can be catalyzed by β-gals from different sources. In addition to microbial-derived β-gals, animal-derived β-gals are also widely used to catalyze the formation of various oligosaccharide units. For instance, β-gal derived from porcine testis, characterized by its ability to preferentially form β-(1→3)-glycosidic linkages, has been used to synthesize the disaccharide Gal-β-(1→3)-GalNAc. The glycans are of significant biological interest, as they are associated with tumor-associated carbohydrate antigens and ABO blood group determinants.

**Fig. 7:**
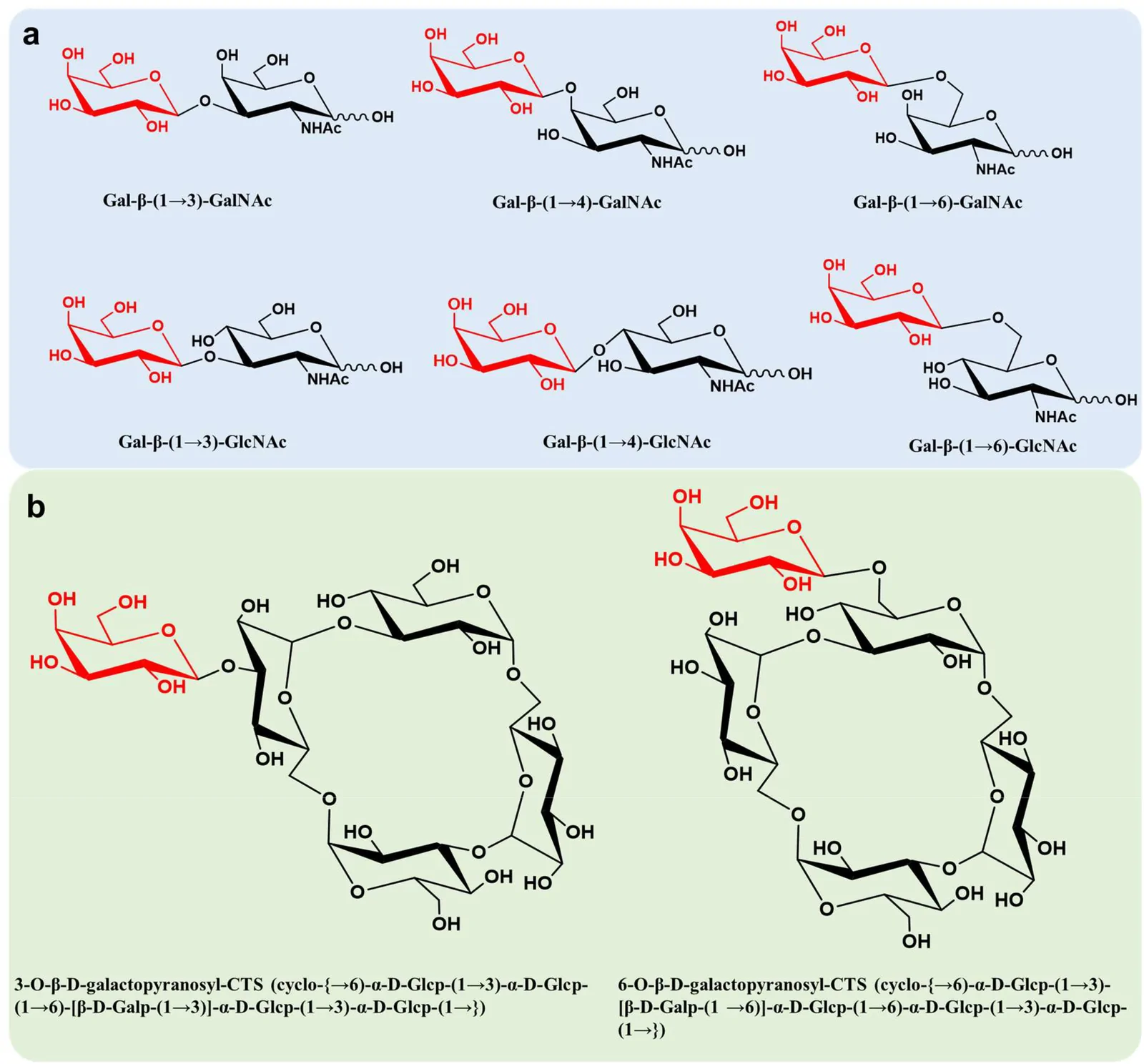
Structures of a series of galactosyl-modified acetylsaminoglycans and galactosyl-CTS. **a**: Structures of galactosyl-modified acetylgalactosamine with different glycosidic bonds. The galactosyl group is red express. **b**: Structures of two galactosyl-CTS.

### 3.4 Glycosylation of carbohydrates with complex structures by β-gal

β-gal not only glycosylates carbohydrate glycosyl acceptors with simple structures but also those with complex structures. Cyclic tetrasaccharide (CTS) has broad application potential as a low-calorie sweetener, a drug-delivery vehicle, and an affinity chromatography material. Given its diverse applications, the enzymatic modification of CTS has attracted interest; studies have shown that β-gals derived from *Aspergillus oryzae* and *Bacillus circulans* can catalyze its conversion to β-D-galactopyranosyl-CTS [75]. The β-gal derived from *Aspergillus oryzae* mainly catalyzes the production of 6-*O*-β-D-galactopyranosyl-CTS, while 3-*O*-β-D-galactopyranosyl-CTS is the main galactosylation product catalyzed by β-gal from *Bacillus circulans*. The structural formulas of the above two products are shown in Fig. 7b.

## 4 Glycosylation of non-carbohydrate acceptors by β-gal

### 4.1 Glycosylation of aliphatic alcohols by β-gal

Simple aliphatic alcohols serve as effective acceptors for β-gal-catalyzed glycosylation. These alcohols can be converted into alkyl glycosides, which have valuable applications in the food and pharmaceutical industries, through transglycosylation [76]. β-gal exhibits absolute stereoselectivity for the anomeric center, along with pronounced regioselectivity toward specific hydroxyl groups. The general reactivity order is primary alcohol > secondary alcohol > phenol > tertiary alcohol. The β-gal-catalyzed reaction process of aliphatic alcohols and the general structural formula of aliphatic alcohols are shown in Fig. 8a. The general structural formula of galactosylated fatty alcohols is shown in Fig. 8b.

**Fig. 8:**
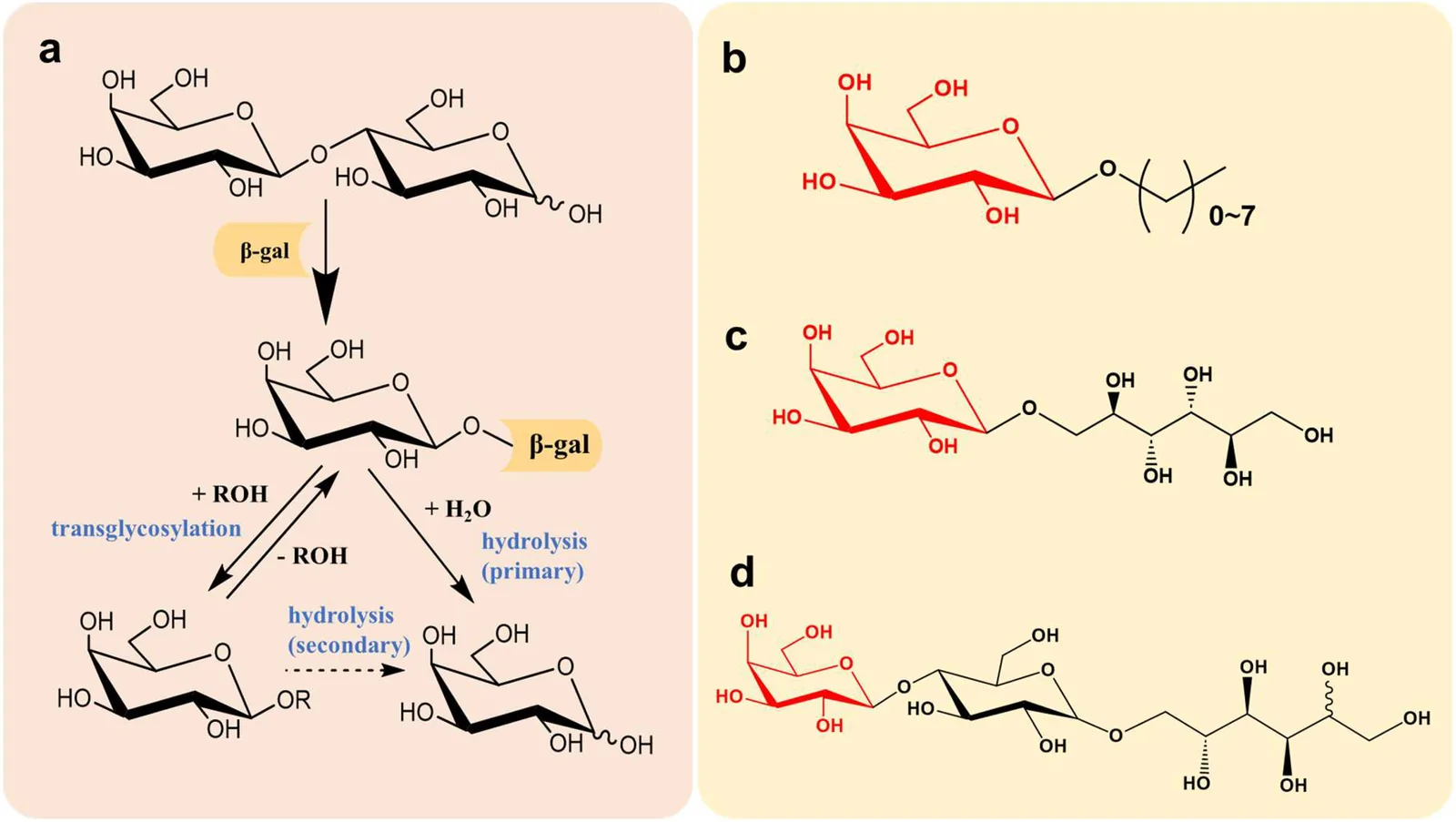
The glycosylation of aliphatic alcohols and sugar alcohols catalyzed by β-gal. **a**: The glycosylation process of aliphatic alcohols and sugar alcohols catalyzed by β-gal. **b**: The structures of aliphatic alcohols. **c**: Galactosylated mannitol (β-D-galactopyranosyl-(1→1′)-D-mannitol). **d**: Galactosylated isomalt (β-D-Galp-(1→3)-α-D-Glcp-(1→6)-D-Glc-ol/Man-ol.

One study has shown that β-gal derived from *Pseudoalteromonas* sp. 22b can catalyze the synthesis of alkyl galactosides and that the number of carbon atoms in the C_3—_C_6_ alcohol molecule has little effect on the product yield [77]. Compared with other mesophilic β-gals, this enzyme can synthesize alkyl galactosides more efficiently when the reaction is carried out in a buffer containing an organic solvent (<50% v/v). Kouptsova et al. [78] demonstrated the possibility of enzymatically synthesizing alkyl glycosides in an aerosol-OT reverse micelle system, which can improve the yield of long-chain alkyl glycosides. Octyl β-D-galactoside can be synthesized from lactose and octanol in one step catalyzed by β-gal. As one of the substrates, octanol can increase the ratio of transglycosylation/hydrolysis and promote transglycosylation. Thus, alkyl glycosides can be obtained from hydrophilic lactose and hydrophobic aliphatic alcohol in one step. The maximum yield of octyl galactoside was 45%, significantly exceeding previously reported yields of enzymatically synthesized alkyl glycosides in a two-phase system. In addition, other studies have demonstrated that β-galactosidase from *Aspergillus oryzae* can be immobilized on a polyethylene glycol-modified gold surface. This system can be used to detect carbohydrate-protein interactions by exploiting the enzyme’s transglycosylation activity to form polysaccharide-protein complex analogs [79]. This chemoenzymatic strategy shows great potential for constructing biomaterials with specific carbohydrate-protein interactions.

### 4.2 Glycosylation of sugar alcohols by β-gal

Mannitol, a sugar alcohol and sorbitol isomer, is an efficient glycosyl acceptor for β-gal. Using *o-*NPG as the donor, a β-gal from *Enterobacter cloacae* B5 catalyzed the synthesis of a novel galactosylated derivative, β-D-galactopyranosyl-(1→1′)-D-mannitol, achieving a yield of 14.6% [80]. The above-mentioned bovine testis-derived β-gal can also synthesize the new β-(1→3)-glycosidic-bond linked GOS derivative β-D-Galp-(1→3)-α-D-Glcp-(1→6)-D-Glc-ol/Man-ol [24] using isomalt as the glycosyl acceptor. The structure of this compound is shown in Fig. 8c and 8d.

### 4.3 β-Gal–catalyzed synthesis of galactosyl prodrugs

β-Gal is also widely used to synthesize galactosyl prodrugs—a strategy in which galactose is covalently linked to an active pharmaceutical ingredient to modify its properties. Galactose is a promising candidate molecule for prodrug design and synthesis because of its nontoxicity, good water solubility, widespread presence of transporters, selective overexpression under pathological conditions, and good chemical properties [81]. Comparing the galactosyl prodrug with the original active pharmaceutical ingredient showed improved solubility, significantly reduced toxicity, and improved targeting.

Doxorubicin is a broad-spectrum antitumor antibiotic drug suitable for the treatment of acute leukemia, malignant lymphoma, and breast cancer. Its toxicity can affect bone marrow hematopoietic function; it also exhibits cardiotoxicity. Huang et al. [82] developed a potential prodrug of doxorubicin with extended-cycle polymerization and pH-triggered drug-release properties. Due to the sustained release of the drug, the cytotoxicity of the prodrug was lower than that of free doxorubicin. Moreover, the galactose-conjugated doxorubicin exhibited enhanced tumor uptake, indicating optimized targeting [82]. The application of galactosyl prodrugs has long been extended to the development of antibiotics and targeted tumor therapeutics. As a powerful tool for catalyzing the synthesis of galactosyl prodrugs, β-gal also has broad application prospects in this field.

#### 4.3.1 Synthesis of antibiotic galactosyl prodrugs

β-Gal can galactosylate some antibiotics containing alcohol groups through a simple reaction**.** A β-gal derived from *Aspergillus oryzae* catalyzes the galactosylation of antibiotic compounds, including chlorophenol and chloramphenicol. One study showed that the yield of galactosylation products was the highest when the enzyme-catalyzed transglycosylation reaction was carried out in 20% (v/v) acetonitrile with lactose as the glycosyl donor [18]. Among them, chlorphenesin or chloramphenicol, which are glycosyl acceptors, only react at their main hydroxyl groups to generate 1-*O*- [β-D-galactopyranosyl]−3- [p-chlorphenoxy]−2‑hydroxy-propanol (CPG) and 3-*O*- [β-D-galactopyranosyl]-D‑threo-2-(dichloracetamido)-1- [p-nitrophenyl]−1-propanol (CAG). The basic reaction process is shown in Fig. 9a. Increasing the concentration of galactosyl donors and receptors can increase the yield of corresponding galactosylated antibiotics. Lee et al. [84] used *E. coli* cells containing β-gal to catalyze the synthesis of galactosylated chlorophenazine. The conversion rate of chlorophenazine to galactosylated chlorophenidin within 12 h reached 64%. Moreover, cytotoxicity experiments confirmed that the galactosylated chlorophenyl produced was significantly less toxic than chlorophenyl.

**Fig. 9:**
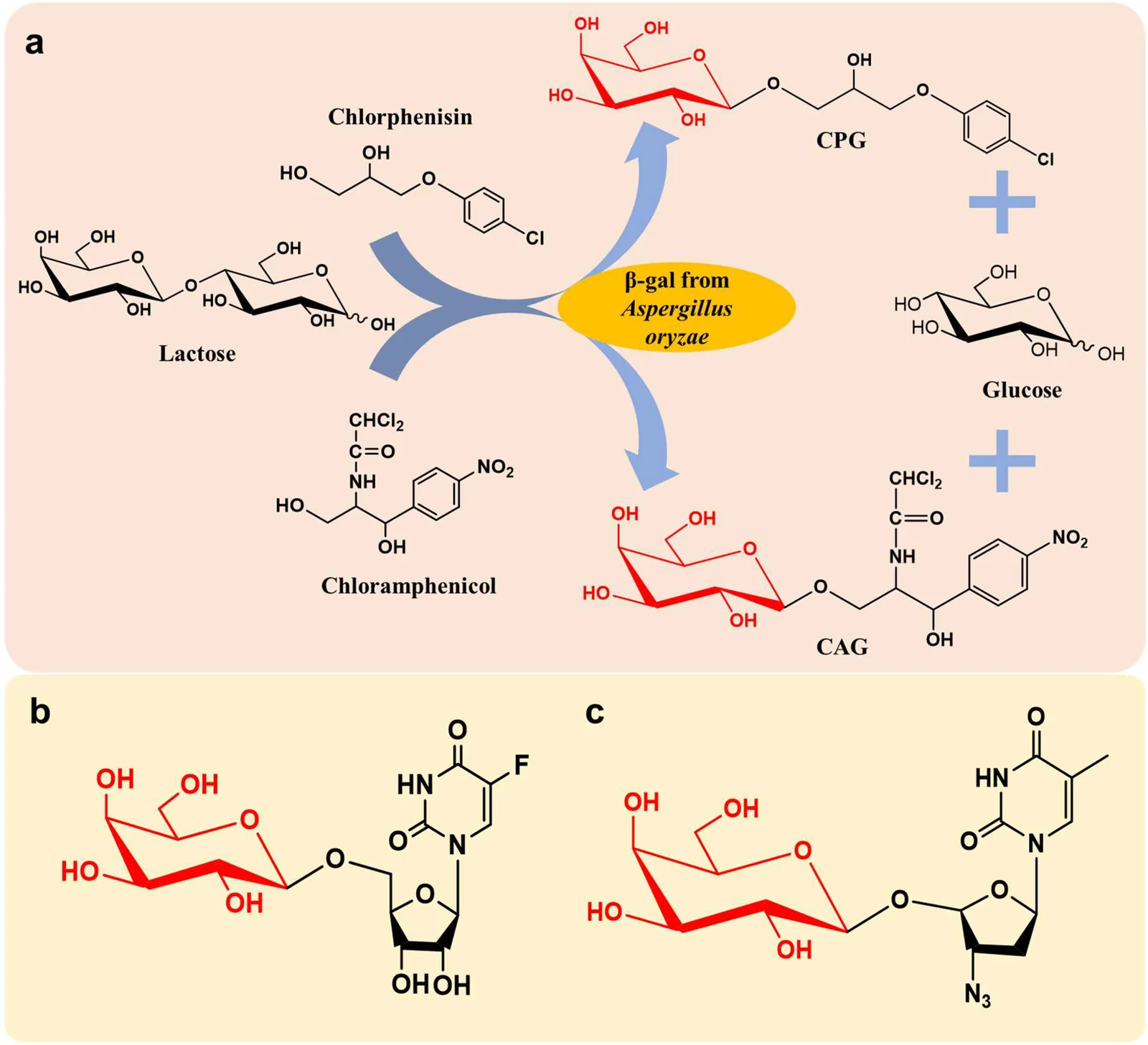
**a**: The reaction of galactosylation of Chlorphenisin and Chloramphenicol catalyzed by β-gal from *Aspergillus oryzae*. **b**: Structures of galactosylated nucleosides galactosylated 5-Fluorouridine (5′-*O*-β-galactosyl-5-fluorouridine). **c**: Structures of galactosylated 3′-Azido-3′-deoxythymidine (5′-O-β-galactosyl-3′-azido-3′-deoxythymidine).

#### 4.3.2 Synthesis of nucleoside galactosyl prodrugs

Nucleoside analogs are widely used in antiviral and anticancer therapy. Studies have shown that many natural antibiotics with antitumor and antiviral activities have nucleoside-oligosaccharide structures [83]. Covalent oligonucleotide-carbohydrate conjugates offer several advantages, including enhanced cellular uptake, improved *in vivo* organ localization, and increased therapeutic efficiency [84]. Synthetic analogs of some natural nucleosides, like β-D-arabinofuranosylcytosine (ara-C), 3′-azido-3′-deoxythymidine (AZT), and 5-fluorouracil-1-β-D-ribofuranoside (5-fluorouridine), are commonly used clinical anticancer or antiviral drugs [85,86]. However, these inhibitors have low selectivity and can kill normal cells as well as tumor cells or virus-infected cells. Therefore, the synthesis of new prodrugs with reduced toxicity has been a research hotspot [87,88].

One study used a conjugate of *E. coli*-derived β-gal and COL-1 monoclonal antibody to release its prodrug from the anticancer prodrug 5′-*O*-β-galactosyl-5-fluorouridine. Cytotoxicity experiments show that the galactosylated prodrug has more than a 100-fold reduction in toxicity to bone marrow cells of BALB/c mice compared with the original drug [89]. Andreotti et al. [84] used a β-gal from *Aplysia fasciata* to catalyze the synthesis of nucleoside galactosyl derivatives with antiviral and antitumor activities. The reaction used *o-*NPG as the glycosyl donor and 5-fluorouridine and the anti-HIV drug AZT as the glycosyl acceptors and synthesized two derivatives: 5′-*O*-β-galactosyl-5-fluorouridine and 5′-*O*-β-galactosyl-3′-azido-3′-deoxythymidine, at yields of 60% and 43%, respectively. All reactions were extremely stereoselective and regioselective, as only 5′-*O*-β-galactosyl conjugates were formed. The synthetic prodrug reactions catalyzed by β-gal mentioned above are highly stereoselective and regioselectivity. As aresult, it is often necessary to screen for β-gals from different sources to identify those that are most active on the chosen glycosyl acceptors. The structures of two nucleoside galactosyl prodrugs are shown in Fig. 9b and 9c.

### 4.4 Synthesis of natural galactosylation products catalyzed by β-gal

#### 4.4.1 Phenolic compounds

Phenolic compounds—including polyphenols such as flavonoids, phenolic acids, lignans, and tannins—represent the most abundant secondary metabolites in plants, vegetables, and fruits. These compounds are characterized structurally by harboring at least one aromatic ring bearing a hydroxyl group [90]. Numerous studies have documented their diverse bioactivities, ranging from antioxidant and cardiovascular-protective to neuroprotective, antiviral, anticancer, antidiabetic, and anti-inflammatory properties [91,92]. Despite these beneficial effects, their poor aqueous solubility results in inadequate stability and bioavailability, thereby limiting their use as nutraceuticals and oral pharmaceuticals [93]. Glycosylation has been shown to improve the water solubility, stability, and bioavailability of phenolic compounds, owing to the hydrophilic nature of sugars that form hydrogen bonds with water via their hydroxyl groups.

This modification can be accomplished via chemical or enzymatic approaches [94]. Enzymatic synthesis is an environmentally friendly method that typically exhibits high regioselectivity. Galactosyltransferases exhibit catalytic promiscuity that favors phenolic galactosylation [[95], [96], [97]]. Plant-derived UDP-galactose-dependent glycosyltransferases have been successfully applied to synthesize phenolic galactosides [95,96,98,99]. However, the high cost of UDP-galactose limits their use. In contrast, β-gal, which uses lactose as an inexpensive, readily available donor, offers a more economical alternative [100,101]. Jesús et al. [90] evaluated the galactosylation capacity of permeabilized cells from two *P. anthophila* strains (Bac 55.2 and Bac 69.1) toward a panel of phenolic compounds with diverse molecular structures. Among the tested substrates, puerarin exhibited the highest conversion rate (up to 53.3% with Bac 69.1), followed by phlorizin and caffeic acid, whereas no reaction was observed for mangiferin. Both strains displayed high catalytic promiscuity toward most phenolic substrates, producing 3 to 7 distinct galactosides. Further kinetic characterization using strain Bac 69.1 revealed its ability to elongate the galactosyl chain, as evidenced by the synthesis of digalactosides from monogalactoside precursors, indicating transgalactosylation activity. The major phenolic galactoside was purified and structurally elucidated via nuclear magnetic resonance, confirming it as a β-D-(1→6)-glycosidic bond-linked puerarin monogalactoside. The catalytic activity of β-gal from *P. anthophila* has been confirmed in the preceding sections. The galactosylation of this series of phenolic compounds benefits precisely from the transglycosylation activity of enzymes produced by *P. anthophila*, of which β-gal is the primary enzyme.

Myricetin, a plant flavonol, has been widely used as an active ingredient in functional foods, medicines, and cosmetics due to its high antioxidant activity. However, because of its aqueous insolubility, its scope of use is severely limited. In order to improve its aqueous solubility, Shimizu et al. [20] used β-gal from *Bacillus circulans* to galactosylate myricetin with lactose as a sugar galactosyl donor. The solubility of the resulting total myricetin-galactoside product is 480 times that of the original myricetin. Four kinds of myricetin-galactosides (the structural formulas of which are shown in Fig. 10a) were then separated from the reaction product via column chromatography. The solubility of these four myricetin-galactosides was >3.9 × 10^3^ times that of myricetin, with each one having an antioxidant activity similar to that of myricetin.

**Fig. 10:**
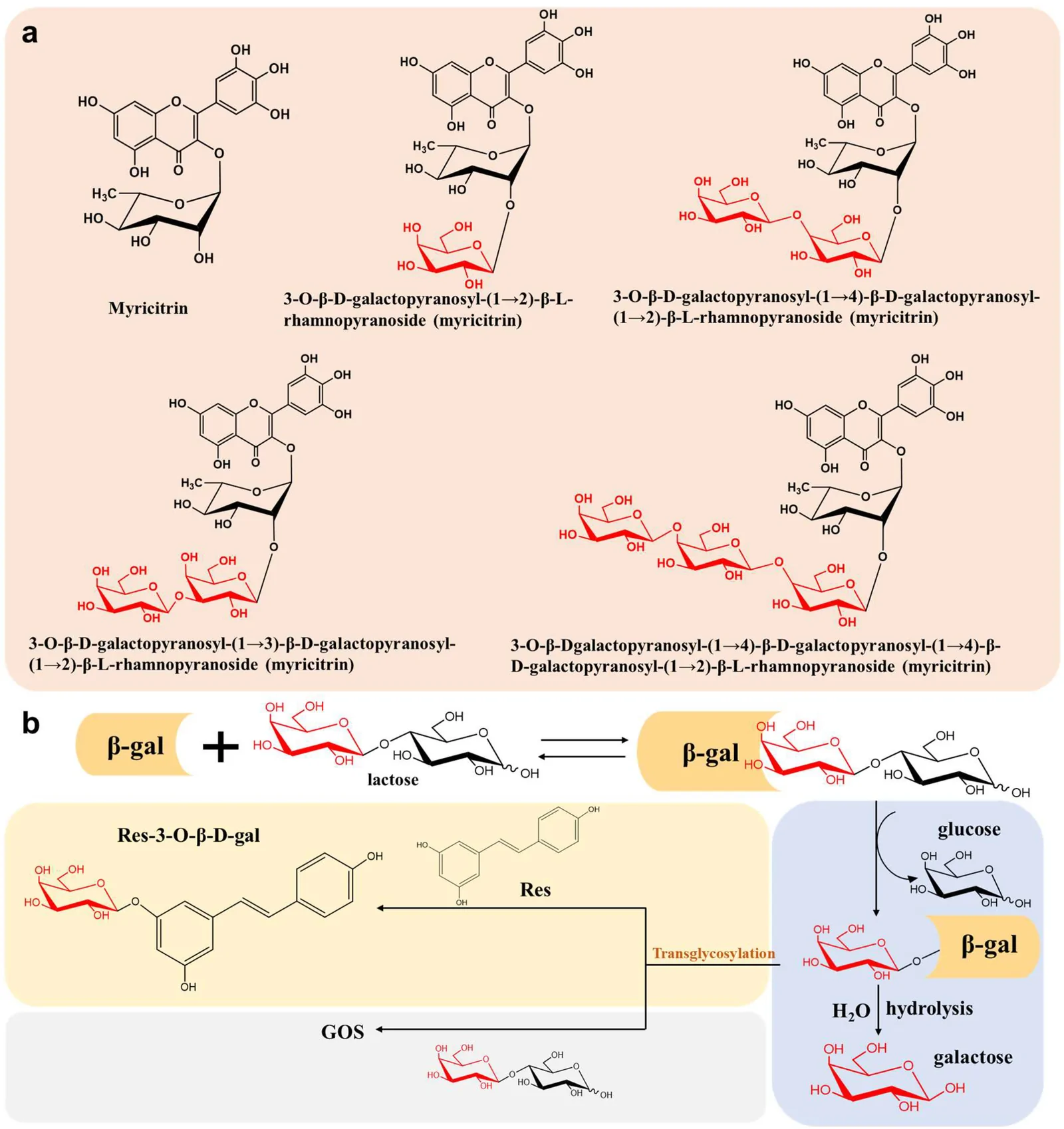
Galactosylation of the natural polyphenolic compounds myricetin and resveratrol. **a**: Structures of myricetin and its galactosylation products. **b**: Synthesis of Res-3-*O*-β-gal catalyzed by β-gal. The yellow module represents the Res-3-*O*-β-gal synthesis reaction; The blue module represents lactose hydrolysis reaction; The gray module represents the GOS synthesis reaction.

Resveratrol (Res) is a natural stilbene polyphenol in plants that can reduce tissue and organ damage caused by various factors. It exhibits various favorable biological activities, including liver protection, inhibition of platelet aggregation, relief of cough and asthma, as well as antibacterial, antiviral, lipid-lowering, and anti-lipid peroxidation effects. These properties demonstrate broad therapeutic potential across various fields, including oncology, neurodegenerative diseases, osteoarthritis, and cardiovascular and metabolic disorders [102]. β-gal derived from *Bacillus megaterium* YZ08 can also catalyze the galactosylation of Res glycosides. This reaction achieved galactosylation modification of the hydroxyl group at position 3-O, producing Res-3-*O*-β-D-gal [103] (Fig. 10b). This study also found that Mg^2+^ can not only improve the stability of β-gal and prolong its half-life but also promote the galactosylation reaction of β-gal in a system comprising 30% DMSO, which is due to the reversal of the hydrolysis thermodynamic equilibrium. In this reaction, the product conversion rate increased by 24–41%.

Astragalin (kaempferol-3-*O*-β-D-glucopyranoside, Ast) is a flavonoid possessing antioxidant, anti-HIV, anti-allergic, and anti-inflammatory properties. However, its therapeutic potential is constrained by its poor water solubility. To overcome this limitation, two novel astragalin galactosides (Ast-Gals) were synthesized via transgalactosylation using a β-gal from *Bacillus circulans*. The optimized reaction conditions yielded kaempferol-3-*O*-β-D-glucopyranosyl-(1→6)-β-D-galactopyranoside and its (1→4)-β-D-galactopyranosyl-extended derivative, both of which demonstrated a > 1300-fold increase in aqueous solubility compared to native astragalin [19]. A β-gal from *Bacillus megaterium* YZ08 demonstrated high catalytic efficiency in synthesizing a galactosylated derivative of the nucleoside analog AZT (3′-azido-3′-deoxythymidine), as well as in catalyzing the galactosylation of naringin, a dihydro-flavonoid compound [103].

#### 4.4.2 Steroidal saponins

Steroidal saponins are important biologically active components of traditional Chinese medicine. They inhibit platelet aggregation and have antibacterial, cytotoxic, and anti-dementia properties. Timosaponin B-II, a furostanol saponin from the rhizomes of *Anemarrhena asphodeloides*, has been shown to improve learning and memory [104]. *L. bulgaricus* L3-derived β-gal catalyzes the galactosylation of four steroidal saponins, including Timosaponin B-II, Polianthoside D, Terrestrinin D and Typaspidoside H1, providing a new enzymatic method for the glycosylation of steroidal saponins [58]. Given that the pharmacological activities of steroidal saponins are highly dependent on their sugar chain composition, there is considerable interest in modifying these glycans to generate compounds with new or enhanced properties. In this context, β-gal-catalyzed galactosylation offers a promising strategy for the structural diversification of steroidal saponins.

### 4.5 β-Gal–catalyzed synthesis of galactosylated amino acid derivatives

Glycopeptides and glycoproteins have irreplaceable recognition roles for structures and molecules in many biological systems, particularly in the specific interactions between glycans and recognition molecules such as lectins, receptors, and antibodies. Moreover, they also have important roles in proteolytic enzyme recognition, intercellular recognition, cell growth, and tumorigenesis. The development of synthetic strategies for the glycosylation of these compounds has important implications for the fields of food, pharmaceuticals, and glycobiology [105,106]. Conjugates of food proteins and carbohydrates can improve certain basic functional properties, such as emulsifying activity, protein solubility, and thermal stability [107]. β-gal generally catalyzes the galactosylation of the hydroxyl group of the hydroxyl‑containing acceptor to generate an *O*-glycosidic bond, so amino acids containing hydroxyl groups, including serine and threonine, can be modified via galactosylation. Seo et al. [110] synthesized a series of galactosylated serine derivatives using β-gal derived from *E. coli*, such as N-carboxy benzyl L‑serine benzyl esters and N‑tert-butoxycarbonyl serine methyl esters. Under the buffer conditions of heptane: sodium phosphate buffer = 70:30 medium, galactosylated N-carboxy benzyl L‑serine benzyl ester with *o-*NPG as the glycosyl donor had the highest yield, up to 23.2%; when lactose was used as the sugar donor, the yield was up to 3.94%. The structures of serine and two galactosylated serine derivatives are shown in Fig. 11.

**Fig. 11:**
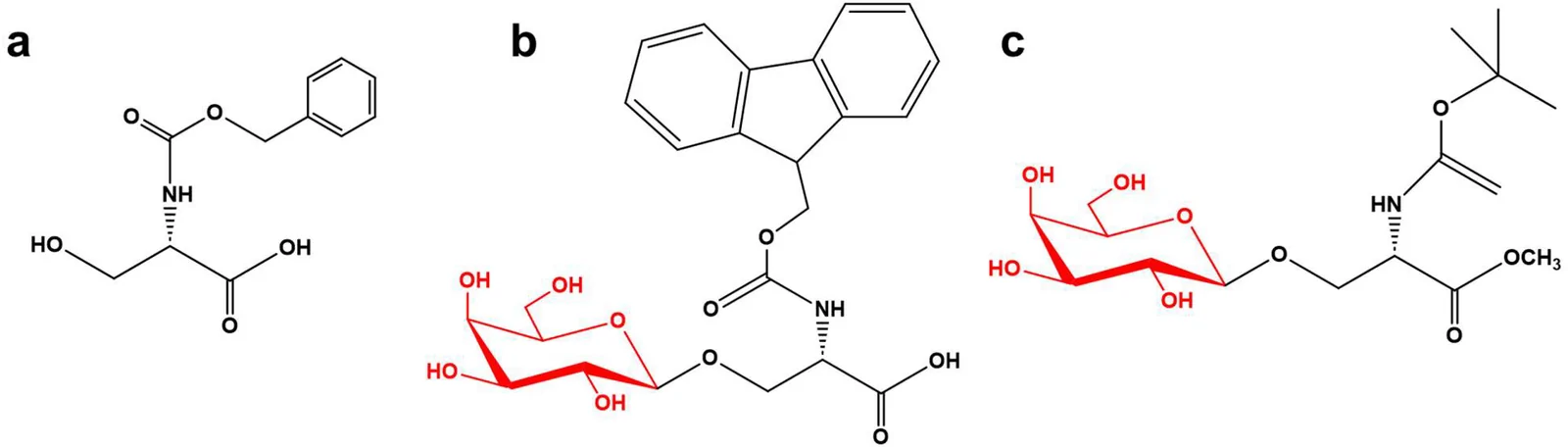
Structures of serine and two galactosylated serine derivatives. **a**: Serine; **b**: Galactosylated N-carboxy benzyl L‑serine benzyl ester; **c**: Galactosylated N‑tert-butoxycarbonyl serine methyl ester.

## 5 Conclusions and prospects

β-gal serves as a powerful tool for galactosylating diverse acceptors without requiring cofactors, enabling the efficient synthesis of GOS and other galactoconjugates. Galactose, owing to its nontoxicity, solubility, and transportability, is an ideal moiety for modifying bioactive compounds to reduce toxicity, enhance targeting, and improve solubility, features that are highly valuable in biopharmaceuticals. Compared to chemical synthesis, enzymatic catalysis offers superior stereoselectivity, regioselectivity, and sustainability while avoiding laborious protecting-group strategies.

The transglycosylation reactions catalyzed by this enzyme exhibit stereoselectivity and regioselectivity, which are highly dependent on the enzyme source. Consequently, the screening and mining of β-gals with desirable properties represent crucial areas of research. Traditional approaches involve isolation from natural environments to establish libraries of highly active glycosidases. Additionally, *in silico* mining from online databases and metagenomic libraries has emerged as an effective strategy for identifying enzymes with superior catalytic performance.

Furthermore, significant progress has been made in the molecular engineering of β-gal, with numerous studies successfully enhancing its transglycosylation activity and substrate tolerance. These engineered variants have expanded the enzyme’s applicability to the glycosylation of structurally complex acceptors. Such advancements are further supported by insights from three-dimensional structural studies and the development of effective high-throughput screening methods to detect transglycosylation activity. Collectively, these efforts provide a robust foundation for the continued utilization and optimization of β-gal across the food, pharmaceutical, and cosmetic industries.

## CRediT authorship contribution statement

**Wei Wei:** Writing – review & editing, Writing – original draft, Methodology, Investigation, Formal analysis, Data curation, Conceptualization. **Jinshan Guo:** Visualization, Validation, Supervision, Resources, Project administration.

## Declaration of competing interest

The authors declare that they have no known competing financial interests or personal relationships that could have appeared to influence the work reported in this paper.
